# Current situation and influencing factors for suicidal intent in patients with intentional acute pesticide poisoning

**DOI:** 10.3389/fpubh.2023.1168176

**Published:** 2023-04-06

**Authors:** Shuang Ma, Zixin Wen, Long Sun, Yingying Zheng, Yanxia Zhang, Longke Shi, Yaqian Li, Guangcai Yu, Jie Zhang, Baotian Kan, Xiangdong Jian

**Affiliations:** ^1^School of Nursing and Rehabilitation, Cheeloo College of Medicine, Shandong University, Jinan, Shandong, China; ^2^Department of Intensive Care Unit, Huashan Hospital, Fudan University, Shanghai, China; ^3^Department of Poisoning and Occupational Diseases, Qilu Hospital, Shandong University, Jinan, Shandong, China; ^4^School of Public Health, Cheeloo College of Medicine, Shandong University, Jinan, Shandong, China; ^5^Department of Sociology, State University of New York Buffalo State, Buffalo, NY, United States; ^6^Department of Geriatric Medicine, Qilu Hospital, Shandong University, Jinan, Shandong, China; ^7^Department of Nursing, Qilu Hospital, Shandong University, Jinan, Shandong, China

**Keywords:** acute pesticide poisoning, cross-sectional survey, intentional, suicidal intent, influencing factors

## Abstract

**Introduction:**

Since pesticides have been widely used in agricultural production, acute pesticide poisoning (APP) has gradually become a worldwide public health problem. Recently, the number of APP cases has been high in China, and the intentional self-administration of pesticides is the main cause of APP. However, there is a lack of relevant studies on the factors influencing suicidal intent in patients with intentional APP. This study aimed to explore the current situation and influencing factors for suicidal intent among patients with intentional APP.

**Methods:**

In this cross-sectional study, we enrolled a total of 225 patients with intentional APP admitted to the emergency department of our Grade A comprehensive hospital in Shandong Province between June 2019 and January 2021. Patients were investigated using a health status interview questionnaire, Beck Suicidal Intent Scale, Duke Social Support Index, psychological stress scale, Dickman Impulsivity Inventory, Trait Anxiety Inventory, Center for Epidemiologic Studies Depression Scale, and Beck Hopelessness Scale. Descriptive statistics, single-factor analysis, and multiple linear regression were used for data analysis.

**Results:**

Suicidal intent scores were collected and averaged (14.23 ± 6.22). Multiple linear regression analysis revealed that marital status, residential area, impulsivity, hopelessness, depression, psychological strain, and social support impact suicidal intent.

**Conclusion:**

Patients with intentional APP have high suicidal intent. Therefore, different interventions should be tailored to different patients.

## 1. Introduction

Since pesticides have been widely used in agricultural production, acute pesticide poisoning (APP) has gradually become a worldwide public health problem (1). Recently, the number of APP cases has been high in China, and the intentional self-administration of pesticides is the main cause of APP (1). In rural developing countries, intentional APP is the preferred method of intentional suicide (2).

In 2017, the World Health Organization (WHO) reported that intentional APP accounts for one-third of suicides worldwide (2). The 2019 edition of the WHO's guidelines on pesticide hazard classification emphasized that banning the use of high-risk pesticides could prevent the deaths of more than 155,000 patients with intentional APP (3). Due to APP's characteristics (which presents as a severe and rapidly changing disease), the mortality rate following APP is high, particularly in developing countries (4). For example, the mortality rate of patients with intentional APP in China is ~6.85% (5). Moreover, intentional APP has imposed a huge economic burden on many individuals and families, resulting in poverty for many rural families and severely affecting the social stability of rural areas (6).

Suicidal intent refers to a person's intent to end their life through suicide. Suicide attempters with high suicidal intent scores have a stronger intention to die than those with low intent scores and may have a higher risk of completing suicide (7).

Many factors, such as age, gender, history of suicide attempts, social support, negative life events, anxiety, depression, impulsivity, and psychological strain, among others, influence suicide intention (8–12). Studies on suicidal intent in Western countries mostly focus on patients with mental disorders, which may be because >90% of suicides in these countries are associated with mental illness (8, 13). We note that there are numerous studies on gender differences among patients with suicidal intent and the relationship between despair, depression, impulsivity, and suicidal intent (9–11). However, only ~30–63% of suicides in China are related to mental illness (14), and there is a lack of relevant studies on the factors influencing suicidal intent in patients with intentional APP.

Therefore, our study (building on previous work on suicidal intent) aimed to describe the suicidal intent of patients with intentional APP within 1 week of hospital admission and comprehensively discuss the influencing factors.

## 2. Materials and methods

### 2.1. Study population and design

Convenience sampling was used to select patients with intentional APP admitted to the emergency department of our Grade A comprehensive hospital in Shandong Province between June 2019 and January 2021. We enrolled patients based on our study inclusion and exclusion criteria.

The study inclusion criteria were as follows: patients with intentional APP (with a clear diagnosis and clear awareness; family members or patients were surveyed regarding medical history and other information); within 1 week of admission; stable vital signs and ability to complete the questionnaire independently or with the help of the researchers; and the provision of informed consent and voluntary participation in this study. Those with language barriers or communication difficulties and those in serious condition were excluded from this study.

Overall, 225 of the eligible participants met the inclusion criteria, and 207 valid questionnaires were completed, with an effective rate of 92.0%.

### 2.2. Measurements

#### 2.2.1. Demographic details

To collect general demographic characteristics, we administered the “Health Condition Interview Questionnaire,” which was constructed by our research group. The main evaluated factors included age, gender, residential area, occupation, marital status, religious belief, mental illness before the suicide attempt, suicide attempt history, family history of suicide, untreated physical disease before the suicide attempt, economic level, family status, and negative life events. The relevance of clinical data to suicidal intent is shown in the Supplementary material.

### 2.3. Primary measures

#### 2.3.1. Suicidal intent

Suicidal intent was measured using the Beck Suicidal Intent Scale (SIS), which is currently popularly administered worldwide (15). There are 15 items on the scale, among which items 1–8 are related to the objective environment comprising the context of the patient's suicidal behavior, and items 9–15 are related to the expectations and perceptions of the suicidal person. Each question is scored on a scale of 0–2. The higher the score, the higher the patient's suicidal intent (that is, the stronger the desire to die). This scale has been widely used in the Chinese population and has demonstrated good validity and reliability (16).

#### 2.3.2. Social support

The Duke Social Support Index (DSSI), developed at Duke University, was also used for evaluation in this study (17). The DSSI comprises the following three subscales: the Social Interaction Subscale (SIS, four items), the Subjective Support Subscale (SSS, seven items), and the Instrumental Social Support Subscale (ISS, 12 items). The SIS and SSS represent Level 3 scores, while the ISS represents Level 2 scores. The sum of the scores of each subscale item represents the score for social support in this dimension, and the total social support score represents the sum of the scores of the three subscales. A higher score indicates a higher level of social support. The Chinese version of DSSI has previously demonstrated high validity and reliability in suicidal individuals (18).

#### 2.3.3. Psychological strain

Zhang (12) developed a mental strain scale to measure mental strain in questionnaire respondents. This scale includes 40 items and four measurement dimensions, each containing 10 items. The questions are scored on a scale of 1–5, ranging from strongly disagree to strongly agree. The higher the score, the more mental strain on a participant. This scale demonstrates good validity and reliability (19).

#### 2.3.4. Impulsivity

The Dickman Impulsivity Inventory (DII) was used to measure personality traits on impulsivity in this study. Dickman categorizes impulses into functional impulsivity (FI) and dysfunctional impulsivity (DI). There are a total of 31 items in the DII. Only the first 23 items were used in this study, with the DI subscale comprising 12 items and the FI subscale comprising 11 items. Each item is graded on a scale of 0–1, with an overall score of 0–23. The higher the scale score, the higher the individual's level of impulsivity. The Chinese version of the DII has demonstrated good validity and reliability in the Chinese population (20).

#### 2.3.5. Anxiety

The Trait Anxiety Inventory used in this study was derived from the State-Trait Anxiety Inventory (STAI) (21). More specifically, the first 20 items are taken from the State Anxiety Inventory, and the last 20 items represent an anxiety trait subscale. The questions are scored on a four-point scale, with an overall maximum score ranging from 20 to 80. The higher the score, the more anxious the individual. A score of <50 is considered normal, a score of 51–60 represents mild anxiety, a score of 61–70 signifies moderate anxiety, and a score of >70 represents severe anxiety. Previous studies have demonstrated that the Chinese version of the STAI is a useful tool for measuring anxiety (22).

#### 2.3.6. Depression

The Center for Epidemiologic Studies Depression Scale (CES-D) was used in this study to evaluate the frequency of depressive symptoms occurring in the past week (23). The scale cannot be used to diagnose clinical depression. Instead, the scale can only be used to evaluate the severity of depressive symptomology. This scale is suitable for use in general population studies. This instrument is scored on a scale of 0–60, and the higher the score, the greater the severity of depressive symptoms. A score of <10 indicates no depression symptomology, a score of 10–20 indicates possible depression, and a score of >20 represents definite depression. The Chinese version of the CES-D has demonstrated good reliability and validity among the Chinese population (24).

#### 2.3.7. Hopelessness

The Beck Hopelessness Scale (BHS) is used to quantify the degree of an individual's negative attitudes toward the future (25). The self-rating scale includes the following three dimensions: feelings about the future, loss of motivation, and expectations about the future. The BHS includes 20 items, and the original scale includes the following two answer options: “true” or “false.” To better reflect interindividual differences, this study extended the score to the following five levels: 1 = complete agreement; 2 = basically consistent; 3 = uncertain; 4 = more or less the opposite; 5 = exactly the opposite. Items 1, 3, 5, 6, 8, 10, 13, 15, and 19 are reverse entries. This instrument is scored on a scale of 20–100; the higher the score, the greater the sense of hopelessness. The Chinese version of the BHS has demonstrated good validity and reliability in previous studies of those attempting suicide (26).

### 2.4. Statistical analysis

SPSS statistical software (v.22.0, Chicago, IL, USA) was used for data entry and statistical analysis. For measurement data, if the data followed a normal or nearly normal distribution and had uniform variance, we generated descriptive data presented as mean ± standard deviation. In addition, *t*-tests were used for comparing means between two groups, and analysis of variance was used for comparing means between more than two groups. Medians and quartile spacing were used for statistical description if the data showed a non-normal or skewed distribution or uneven variances.

Non-parametric Wilcoxon rank-sum and Kruskal–Wallis *H*-tests were used for generating statistical inferences. The classification variables were described statistically as frequencies and percentages. Furthermore, correlation analysis was used to evaluate the correlations between variables. Pearson correlation analysis was used for bivariate, normally distributed data. Spearman rank correlation was used for the statistical analysis of non-normally distributed data. Continuous normally distributed data and multiple linear regression were used for regression analysis. Statistical significance was set at *P* < 0.05.

## 3. Results

### 3.1. General demographic information

General demographic information and a single-factor analysis for the 207 patients with intentional APP included in this study are presented in Table 1. The results of the univariate analysis showed that age, residential area, marital status, occupation, history of attempted suicide, mental illness before intentional APP, economic status, and family status had statistically significant effects on the instrument-derived scores of the patients with intentional APP (*P* < 0.05).

**Table 1:** Demographic information and single-factor analysis in patients with intentional acute pesticide poisoning (*n* = 207).

	**Number (*n*)**	**Percentage (%)**	**SIS score**	***t*/*F***	***P***
**Gender**				0.01	0.992
Male	93	44.9	14.24 ± 6.25		
Female	114	55.1	14.23 ± 6.23		
**Age (years)**				6.925	0.001
12–34	106	51.2	15.44 ± 6.44^#^		
35–54	64	30.9	11.92 ± 5.84*		
55–81	37	17.9	14.76 ± 5.20^#^		
**Region**				−2.379	0.018
Country	183	88.4	13.86 ± 5.96		
City	24	11.6	17.04 ± 7.52		
**Education**				1.938	0.106
Illiteracy	14	6.8	13.09 ± 6.27		
Primary school	53	25.6	13.72 ± 6.63		
Middle school	83	40.1	15.29 ± 5.61		
High school	41	19.8	17.31 ± 6.19		
University	16	7.7	14.93 ± 3.99		
**Marital status**				3.869	<0.001
Unmarried	58	28.0	16.83 ± 6.09		
Married and “other”	149	72.0	13.22 ± 6.00		
**Occupation**				2.687	0.032
Peasant	60	29.0	12.72 ± 5.94^#^		
Individual business	29	14.0	13.62 ± 6.10^#^		
Worker	40	19.3	14.55 ± 6.35		
Student	24	11.6	17.42 ± 5.93*		
Other	54	26.1	14.59 ± 6.25		
**Religion**				1.350	0.179
Yes	20	9.7	12.45 ± 5.30		
No	187	90.3	14.42 ± 6.30		
**History of attempted suicide**				3.755	<0.001
Yes	33	15.9	17.85 ± 5.34		
No	174	84.1	13.55 ± 6.16		
**Mental illness**				3.520	0.001
Yes	45	21.7	17.04 ± 5.60		
No	162	78.3	13.45 ± 6.18		
**Economic level**				2.129	0.034
Below average	59	28.5	15.68 ± 5.82		
Medium and above	148	71.5	13.66 ± 6.31		
**Family status**				3.122	0.046
Higher	103	50.5	13.24 ± 6.03		
Normal/average	90	44.2	14.97 ± 6.32		
Lower	11	5.3	17.09 ± 6.67		
**Living alone**				1.003	0.317
Yes	33	16.4	15.27 ± 6.01		
No	168	83.6	14.08 ± 6.30		
**Somatopathy**				−0.223	0.824
Yes	49	23.7	13.78 ± 4.99		
No	158	76.3	14.25 ± 6.28		
**Family history of suicide**				0.804	0.442
Yes	9	4.3	14.86 ± 4.56		
No	198	95.7	14.04 ± 6.58		

SIS, Beck Suicidal Intent Scale. Compare pairwise with^#^, ^*^P < 0.05.

### 3.2. Suicidal intent

The average suicidal intent score for patients with intentional APP was 14.23 ± 6.22. In the objective portion of the study, the average score for the help-seeking behavior item was the highest (1.55 ± 0.77); 72.0% of the patients with intentional APP did not contact or inform those who might help them before acting. In the subjective portion of the study, the score for patients' attitudes toward the suicide attempt was the highest (1.50 ± 0.68); 60.4% of the patients with intentional APP were serious about ending their own lives, as presented in Tables 2, 3.

**Table 2:** Suicidal intent scores in patients with intentional acute pesticide poisoning (*n* = 207).

**Entry**	**Score**	**Rank**
Suicidal intent	14.23 ± 6.22	
**Objective questions**		
Isolation	1.38 ± 0.76	2
Possibility of intervention	1.24 ± 0.83	3
Degree of prevention	0.87 ± 0.80	4
Help-seeking behavior	1.55 ± 0.77	1
Arrangements after death	0.59 ± 0.76	6
Active preparations	0.77 ± 0.79	5
Last words or notes	0.35 ± 0.63	7
References to suicide attempts	0.31 ± 0.61	8
**Subjective questions**		
Purpose of suicide	1.34 ± 0.72	3
Expectation of death	0.99 ± 0.95	4
Lethality of the suicide method	0.68 ± 0.86	5
Attitude toward the suicide attempt	1.50 ± 0.68	1
Desire to live or die	1.39 ± 0.78	2
Likelihood of being saved	0.68 ± 0.79	5
Degree of consideration	0.61 ± 0.85	6

**Table 3:** Help-seeking behavior and attitude toward the suicide attempt in patients with acute pesticide poisoning (*n* = 207).

**Entry**		**Number (*n*)**	**Percentage (%)**
Help-seeking behavior	Informing others of a suicide attempt	36	17.4
	Contacting someone but not informing them about the suicide attempt	22	10.6
	No contact with others	149	72.0
Attitude toward the suicide attempt	No intent for death to occur	22	10.6
	Not sure	60	29.0
	Active intent for death to occur	125	60.4

### 3.3. Influence of psychosocial factors

We found a positive correlation between suicidal intent and psychological strain, hopelessness, anxiety, and depression in patients with intentional APP. The suicidal intent of patients with intentional APP was negatively correlated with impulsivity and social support, as presented in Table 4.

**Table 4:** Correlation analysis for the association between the social support score in patients with intentional acute pesticide poisoning and suicide intentionality (*n* = 207).

	**Score (M ±SD)**	***t*/*r***	***P***
**Negative life events**		1.021	0.308
Yes	14.40 ± 6.25		
No	13.04 ± 6.02		
DSSI	34.63 ± 6.50	−0.342	<0.001
SIS	8.15 ± 2.26	−0.341	<0.001
SSS	16.31 ± 3.24	−0.235	0.001
ISS	10.16 ± 2.52	−0.307	<0.001
Psychological strain	118.28 ± 19.08	0.179	0.010
Wish strain	28.72 ± 7.72	0.238	0.001
Value strain	31.10 ± 3.93	0.144	0.038
Relative deprivation strain	25.40 ± 8.27	0.315	<0.001
Coping strain	31.46 ± 7.08	0.285	<0.001
Hopelessness	56.75 ± 11.72	0.356	<0.001
Impulsivity	10.28 ± 4.82	−0.207	0.003
Functional impulsivity	4.99 ± 3.08	−0.196	0.005
Dysfunctional impulsivity	5.29 ± 2.77	−0.143	0.039
Anxiety	47.66 ± 5.30	0.294	<0.001
Depression	24.88 ± 9.92	0.327	<0.001

DSSI, Duke Social Support Index; ISS, Instrumental Social Support Subscale; SD, standard deviation; SIS, Beck Suicidal Intent Scale; SSS, Subjective Support Subscale.

### 3.4. Multivariate analysis evaluating suicidal intent

Multiple linear regression was conducted, with the suicidal intent of patients with intentional APP defined as the dependent variable and variables showing statistically significant differences on single-factor analysis (from among general sociodemographic, sociological, and psychological factors) defined as the independent variables.

A stepwise independent variable screening method was also conducted. The criterion for variable elimination was a *P*-value corresponding to an F value. The statistical significance levels of the entry and elimination variables were *P* ≤ 0.10 and *P* ≥ 0.15, respectively. Marital status, region of residence, impulsivity, hopelessness, depression, psychological strain, and social support were entered into the regression equation, with the following values assigned to the marital status and residential area variables: married and “other” = 1, never married = 0; rural = 1, urban = 0). The regression model was statistically significant *F* = 11.505 (*P* < 0.001; Table 5).

**Table 5:** Multiple linear regression analysis evaluating influencing factors for suicidal intent (*n* = 207).

	***B***	**Sb**	***b*'**	***T***	**95% CI**	***P***
Constant	20.974	4.803		4.367	11.503 to 30.445	<0.001
Marital status	−2.239	0.868	−0.162	−2.579	−3.952 to −0.527	0.011
Region	−2.240	1.172	−0.116	−1.912	−4.551 to 0.070	0.057
Impulsivity	−0.248	0.084	−0.196	−2.947	−0.415 to −0.082	0.004
Hopelessness	0.142	0.037	0.267	3.828	0.069 to 0.215	<0.001
Depression	0.130	0.048	0.207	2.695	0.035 to 0.225	0.008
Psychological strain	0.051	0.028	0.157	1.885	0.003 to 0.106	0.065
Social support	−0.173	0.068	−0.181	−2.542	−0.307 to −0.039	0.012

CI, confidence interval.

R = 0.537, F = 11.505, P < 0.001.

## 4. Discussion

### 4.1. Suicidal intent

In our study, the average score for suicidal intent in patients with intentional APP was considerably higher than the scores for suicidal intent in individuals who made serious suicide attempts in rural China, as documented in previous studies (27). The total average score for suicidal intent in patients with intentional APP was also lower than the score for suicidal intent reported in a previous study by Tong et al. but was higher than the score for suicidal intent in patients who attempted suicide in that study (28). In the same study, more than half of those attempting suicide took pesticides; however, the intensity of suicidal intent in the pesticide users was lowest regardless of the endpoint of death or attempted suicide. Even in the group that had attempted suicide, the score was lower than in those using drugs to attempt suicide; this was inconsistent with the results of our study. The underlying reason may be that the patients admitted to the hospital selected in our study were in critical condition and that the number of patients taking poison was large. Furthermore, we found that the amount of poison taken was highly correlated with the intensity of the patient's suicidal intent, while no detailed clinical data have been reported in previous studies; this may represent one of the reasons for the observed differences in results between our study and previous work.

Moreover, our study showed that 72.0% of the patients with intentional APP did not contact or inform those who could help them before taking action, and that 60.4% of the patients with intentional APP were serious about wanting to end their own life. This is consistent with the results of previous studies among rural suicide attempters. For example, another study in China reported that only 39% of migrant workers sought help when needed, compared with 67% of urban residents (and 86% of non-migrant rural residents). Another study on Australian adults showed that 36.5% of the study participants were likely to seek help from anyone for suicidal thoughts (29), while many other studies have reported a higher prevalence of seeking help (30, 31). Some researchers have suggested that this may be due to the differential stigmatization of suicide in various cultures and populations (32). Chinese researchers have suggested that suicide is more stigmatized in China than in other countries due to the influence of Confucian culture. Since the Chinese are ashamed of suicide, fewer of those who may be experiencing suicidal ideation seek help before committing suicide.

### 4.2. Factors influencing suicidal intent

#### 4.2.1. Demographic factors

In a previous study of rural suicide attempters in China, marriage was not found to be a protective factor for suicide (33). However, the study found that marriage was a protective factor for suicide attempts, with those who were never married demonstrating a higher degree of suicide attempts than those with a married or “other” (i.e., divorced, widowed, or separated) marital status, which is inconsistent with the results of previous research.

Previous studies have shown that age is a factor affecting suicidal intention. For example, a Spanish study (34) showed that suicidal intention decreased with the increase in age, and young people had higher suicidal intentions than older people. This is inconsistent with the results of this study. However, a study of attempted suicide in China concluded that advanced age could not be considered a risk factor for suicidal intent (35). The underlying reason may be associated with the age of the enrolled population. For example, studies enrolling younger students or school dropouts may show different results, as younger individuals generally have a less established value system and a reduced ability to withstand suffering. Younger individuals experiencing learning disabilities, higher academic pressure, conflicts with classmates, and fierce clashes with parents may cloud results for the many younger individuals with mental disorders enrolled in these studies. In contrast, for somewhat older unmarried men in rural areas, it is frequently difficult or impossible to find a suitable partner or even to pray for a happy marriage. Due to pressure to marry and the stigma and disappointment of failed relationships, many cannot withstand family and peer pressure, which may lead to a higher degree of suicidal intent (including in patients with mental illness).

In previous studies, the higher suicidal intention was frequently associated with mental disorders, and suicide attempts with mental illness have higher suicidal intention (36–38). Eddleston found that 40% of the pesticide group was diagnosed with mental disorders (39). Although the prevalence rate of mental illness diagnosis in this study was relatively low (21.7%), univariate analysis showed that the suicidal intention of patients with untreated mental illness before APP was higher than that of those without mental illness.

In a study, patients with intentional APP who were living in cities showed a higher degree of suicidal intent than those living in rural areas, which is inconsistent with previous research findings (28). Previous studies have speculated that suicide attempters living in rural areas may be more likely to take pesticides due to a lower degree of education, low-income family economic status, and various other reasons than those living in urban areas. Additional contributing factors may be that, barring an interest in purchasing pesticides online or through other means, access to pesticides is lower among urban dwellers; therefore, impulsive suicide attempts using pesticides are certainly less likely.

#### 4.2.2. Social support

The study shows that social support is a protective factor for suicidal intent in patients with intentional APP. The higher the level of social support, the lower the level of suicidal intention. This is consistent with the findings of previous research (conducted both domestically and abroad) (40–42). Social support (particularly from family members) provides a support system that can effectively alleviate the psychological or spiritual pain caused by negative life events, thereby meaningfully reducing suicidal intent even in adverse circumstances. A study by Wilcox et al. reported that a low level of social support is a risk factor for persistent suicidal ideation and a predictor of suicide. Cross-cultural studies with large samples have also suggested that a high degree of social support is associated with a low suicide rate and that social support can be used as a clear means of suicide intervention (43).

#### 4.2.3. Hopelessness, anxiety, depression, psychological strain, and impulsivity

This study found that patients with intentional APP had a higher incidence of anxiety and depression. This was higher in patients with mental disorders but without severe suicide attempts in a previous investigation conducted in rural China but lower in those with mental disorders and severe suicide attempts (28). Psychological problems in patients with intentional APP deserve attention. The results of the multivariate analysis employed in this study showed that despair was a risk factor for suicidal intent in patients with intentional APP. Moreover, many studies have shown that despair is one of the best predictors of suicide (44, 45), which is also consistent with the results of our study. Underlying reasons may be that people with a higher level of despair tend to be world-weary, lose enthusiasm and confidence in life, and have no hope for the future, thereby increasing their risk of suicidal behavior and leading to a higher level of suicidal intent. In our study, anxiety and depression were risk factors for suicidal intent in patients with intentional APP. Previous studies have demonstrated that suicide attempters with anxiety and depression have stronger suicidal intent (46), which is also consistent with the results of our study.

The results of the single-factor analysis employed in this study showed a correlation between suicidal intent and value strain, wish strain, relative deprivation strain, and coping strain in patients with intentional APP. The results of the multifactor analysis showed that psychological strain still had a predictive effect on suicidal intent when controlling for other relevant factors and that the higher the degree of psychological strain, the stronger the suicidal intent. This was consistent with the findings of the study by Zhang et al. (33) Our study further confirmed that psychological strain also showed a statistically significant predictive effect on suicidal intent in patients with intentional APP.

We have observed that the strain theory of suicide holds that strain exists before the suicide and has a meaningful predictive effect on suicidal behavior. This type of individual strain before suicide has been documented in suicide notes written by American suicide attempters (47). When torque-to-introverted aggression occurs, suicide will occur. In contrast, torque-to-extroverted aggression can lead to criminal behavior against others (48). Therefore, when psychological strain reaches a certain level, we should pay attention and adopt solutions to reduce psychological strain to prevent the occurrence of suicidal or criminal behavior.

Previous studies suggest that a large portion of suicide deaths and suicide attempts in China are impulsive behavior with weak suicidal intentionality, and the characteristics of these individuals are meaningfully different from those with strong suicidal intent (16). In this study, the multifactor analysis showed that impulsivity was a protective factor for suicidal intent in patients with intentional APP, further verifying that intentional APP is a characteristic form of impulsive suicide. A high level of impulsivity, frequently without careful consideration and occurring immediately before the implementation of suicidal behavior, is intuitively associated with lower suicide intentionality.

### 4.3. Strengths and limitations

Previous relevant studies evaluating influencing factors for suicide intentionality have mostly focused on those attempting suicide in rural areas. This study conducted an in-depth analysis of suicidal intent in patients with intentional APP based on clinical data and completed the investigation within 1 week after the occurrence of the suicidal behavior. Moreover, this study evaluated more than 20 relevant factors, including those on demographics, sociology, psychology, and biology. This is conducive to our screening of factors related to suicidal intent in patients with intentional APP to provide a comprehensive and substantive reference for clinical intervention.

However, in addition to these substantial strengths, this study had some notable limitations. First, regarding the sampling methodology, convenience sampling was adopted instead of strictly following the principle of random sampling, which may have led to an inaccurate description of the epidemiological status of APP (i.e., differing from the overall status in the country). Second, this study focused on screening key risk factors without an in-depth discussion of the possible relationships between each factor. Therefore, future research should further explore the relationships between variables and raise the level of research on risk factors to theoretical construction. Third, considering the actual situation, the psychological variables included in this study are not significantly comprehensive, which will be further improved and supplemented in the future.

## 5. Conclusions

We found a relatively high level of suicidal intent in the population of patients with intentional APP. Unmarried patients living in cities with high levels of depression, despair, and psychological tension merit appropriate psychological interventions and physical treatment to promote recovery and reduce the incidence of repeated suicide. Therefore, different interventions should be implemented for patients with impulsive suicide attempts and strong social support.

## Data availability statement

The original contributions presented in the study are included in the article/Supplementary material, further inquiries can be directed to the corresponding authors.

## Ethics statement

The studies involving human participants were reviewed and approved by the Ethics Committee of Qilu Hospital of Shandong University. The patients/participants provided their written informed consent to participate in this study.

## Author contributions

SM, JZ, and BK conceived and designed the study. SM, LSu, YZhe, YZha, LSh, YL, and GY collected and analyzed data. SM and ZW drafted the paper. JZ, BK, and XJ reviewed and edited the manuscript. All authors read and approved the manuscript and agree to be accountable for all aspects of the research in ensuring that the accuracy or integrity of any part of the work is appropriately investigated and resolved.
