# Supplementary material for: Current situation and influencing factors for suicidal intent in patients with intentional acute pesticide poisoning
Source: Front Public Health. 2023 Apr 6;11:1168176. doi: 10.3389/fpubh.2023.1168176 (PMC10117821; doi:10.3389/fpubh.2023.1168176)
Supplement: Supplementary file 1 [file Table_1.DOCX]

Supplementary Material

Current Situation and Influencing Factors for Suicidal Intent in Patients with Intentional Acute Pesticide Poisoning

Shuang Ma^1,2†^, Zixin Wen^1,3†^, Long Sun^4^, Yingying Zheng^1,3^, Yanxia Zhang^1,3^, Longke Shi^3,4^, Yaqian Li^3^, Guangcai Yu^3^, Jie Zhang^4,5*^, Baotian Kan^1,6*^, Xiangdong Jian^3,4*^

*** Correspondence:** Xiangdong Jian, PhD; E-mail: jianxiangdongvip@vip.163.com

**Correlation between clinical data and suicidal intentions**

# Method

Clinical information mainly included pesticide type, harmfulness, the dosage of poison taken, and type of mixed-use of pesticides, and the data were obtained from clinical medical record diagnosis.

Common clinical pesticides are mainly categorized into herbicides, insecticides, and rodenticides. In a study with few mixed pesticides with more than two types, pesticide types are categorized into one type and two or more. The dose of poison was classified into a group of 30 mL. When the dose of poison exceeded 150 mL, the number of people began to decrease. Therefore, the group with a small number of people was combined. The toxicity classification of pesticides is mainly based on the Guidelines for Classification of Pesticide Hazards recommended by the World Health Organization (2019 edition) (1). LD_50_ of pesticides is taken as an indicator to measure the toxicity of pesticides. Tests are conducted on rats to determine pesticides' oral LD50 and skin contact LD50. Pesticides can be classified into the following five levels according to the median lethal dose of pesticides: extreme risk, high risk, moderate risk, mild risk, and unlikely acute risk (Table 1). This study classified and integrated the highly lethal pesticides based on the reference guidelines and combined with the consensus of clinical experts on different pesticide poisoning. Paraquat is a bipyridine compound with the formula C12H14N2C12 and the LD_50_ of rat intragastric administration of 150 mg/kg. Although it is classified as moderately toxic in toxicology, it is clinically classified as highly toxic (extreme risk) due to its high fatality rate [131]. There is a phenomenon of mixing Aquacide with paraquat in the Chinese pesticide market [132], and according to clinical poisoning treatment experience, the case fatality rate of oral Aquacide with paraquat is higher than that of simple Aquacide poisoning; therefore, this study classified Aquacide with paraquat as extremely risk. As mild risk and unlikely acute risk accounted for less in this study, it was combined with moderate risk as "moderate risk and other."

**Table 1. Classification of pesticide hazards recommended by the WHO**

| Classification |  | Rat LD_50_  mg/kg | |
| --- | --- | --- | --- |
|  |  | Oral administration | Skin exposure |
| Ⅰa | Extreme risk | <5 | <50 |
| Ⅰb | High risk | 5–50 | 50–200 |
| Ⅱ | Moderate risk | 50–2000 | 200–2000 |
| Ⅲ | Mild risk | >2000 | >2000 |
| U | Unlikely acute risk | >5000 |  |

# WHO, World Health Organization

# Result

The clinical data and univariate analysis of patients with acute pesticide poisoning (APP) intention included in this study are presented in Table 2. Patients with intentional APP who took a combination of pesticides scored higher on suicidal intention than those with intentional APP who used only one pesticide (P<0·05). No significant difference was found in the suicidal intention of patients with intentional APP among pesticide types and pesticide harmfulness groups (P>0·05). The amount of poison taken by intentional APP patients was positively correlated with suicidal intention (r=0·551, P<0·001).

**Table 2. Clinical information and single-factor analysis in patients with intentional acute pesticide poisoning**

|  | Number (n) | Percentage (%) | SIS score | t/F/r | P |
| --- | --- | --- | --- | --- | --- |
| Pesticide type | 201 |  |  | 0·983 | 0·402 |
| Herbicide | 130 | 64·6 | 13·91±6·11 |  |  |
| Insecticides | 36 | 18·0 | 13·83±6·04 |  |  |
| Rodenticides | 8 | 4·0 | 14·57±7·61 |  |  |
| Other | 27 | 13·4 | 16·07±6·13 |  |  |
| Pesticide type | 200 |  |  | -2·630 | 0·009 |
| 1 | 182 | 91·0 | 13·81±6·29 |  |  |
| >1 | 18 | 9·0 | 17·83±5·00 |  |  |
| Harmfulness of pesticide | 170 |  |  | 2·261 | 0·107 |
| Ⅰa (Extreme risk) | 97 | 57·1 | 13·38±6·00 |  |  |
| Ⅰb (High risk) | 16 | 9·4 | 13·06±6·02 |  |  |
| Ⅱ (Moderate risk and others) | 57 | 33·5 | 15·42±6·18 |  |  |
| Dose of poison |  |  |  | 0·551 | <0·001 |

# Conclusion

Univariate analysis showed that there were statistical differences in the effects of the dosage of poison and pesticide mix on the suicidal intention of patients with intentional APP (P<0·05); the higher the dosage of poison, the higher the suicidal intention score (P< 0·001), and patients taking a combination of pesticides were more likely to attempt suicide than those taking only one pesticide (P=0·021).

# References

1.WHO recommended classification of pesticides by hazard and guidelines to classification, 2019 edition. Geneva: World Health Organization; 2020.

2.Paraquat poisoning diagnosis and treatment "Taishan consensus" expert group, Xiangdong Jian. "Taishan consensus" on diagnosis and treatment of paraquat poisoning [J]. Chin J Industrial Medicine, 2014, 27(02): 117-119. (In Chinese)

3.Yu Dong. Paraquat disguises low price Diphtheria to return to the market [N]. Farmers Daily, 2017-06-22 (006). (In Chinese)
